# Supplementary material for: The impact of anthropogenic land use and land cover change on regional climate extremes
Source: Nat Commun. 2017 Oct 20;8:989. doi: 10.1038/s41467-017-01038-w (PMC5651924; doi:10.1038/s41467-017-01038-w)
Supplement: Supplementary file 1 — Supplementary Information [file 41467_2017_1038_MOESM1_ESM.pdf]

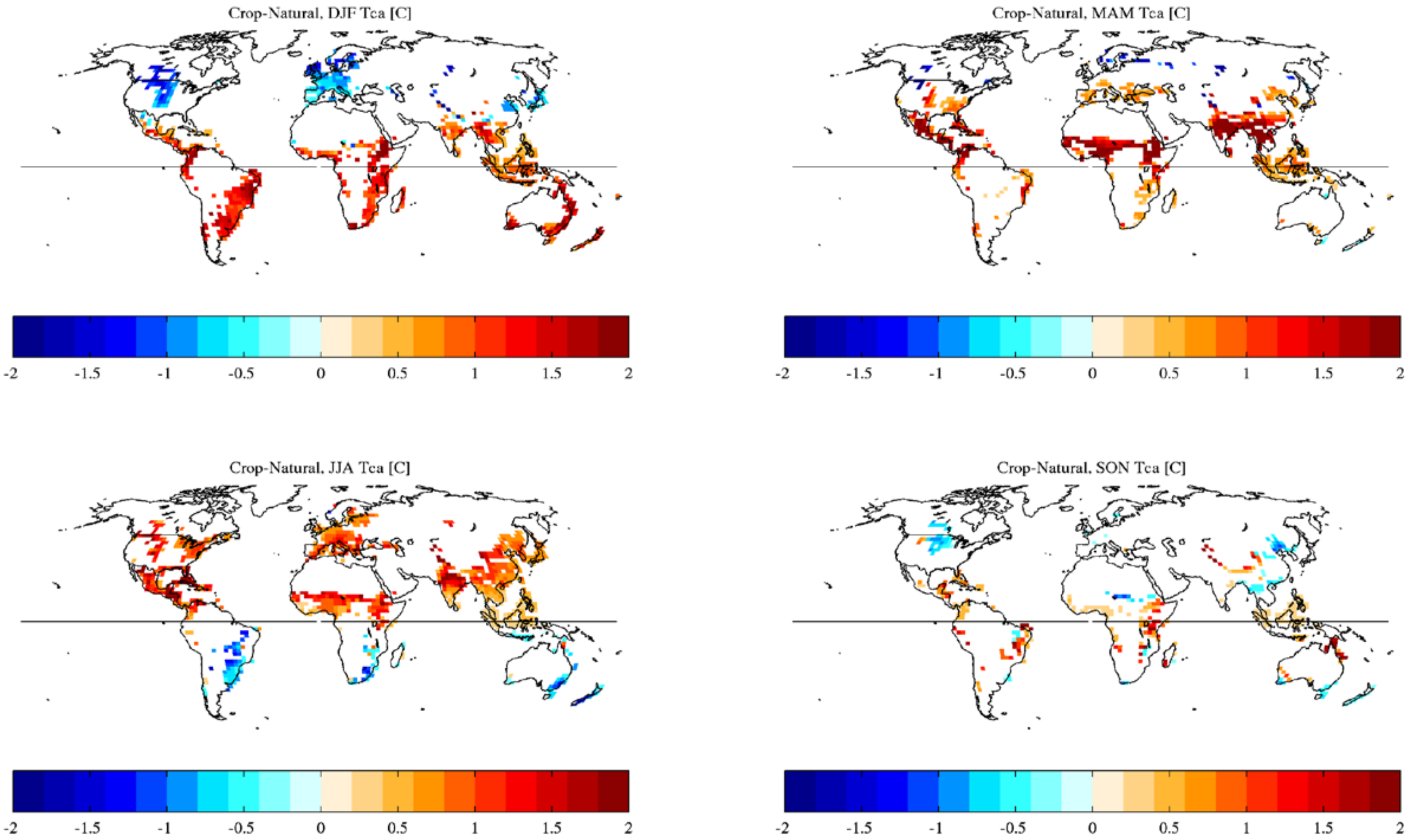

Supplementary Figure 1: Seasonal differences in canopy air temperature between crop tiles and natural tiles in the AllHist runs averaged over the 1981-2005 time period (˚C). Differences computed in grid cells with at least 5% of the grid cell as crop. Differences shown pass a modified t-test at the 90% significance level. DJF for December-February; MAM for March-May; JJA for June-August; SON for September-November.

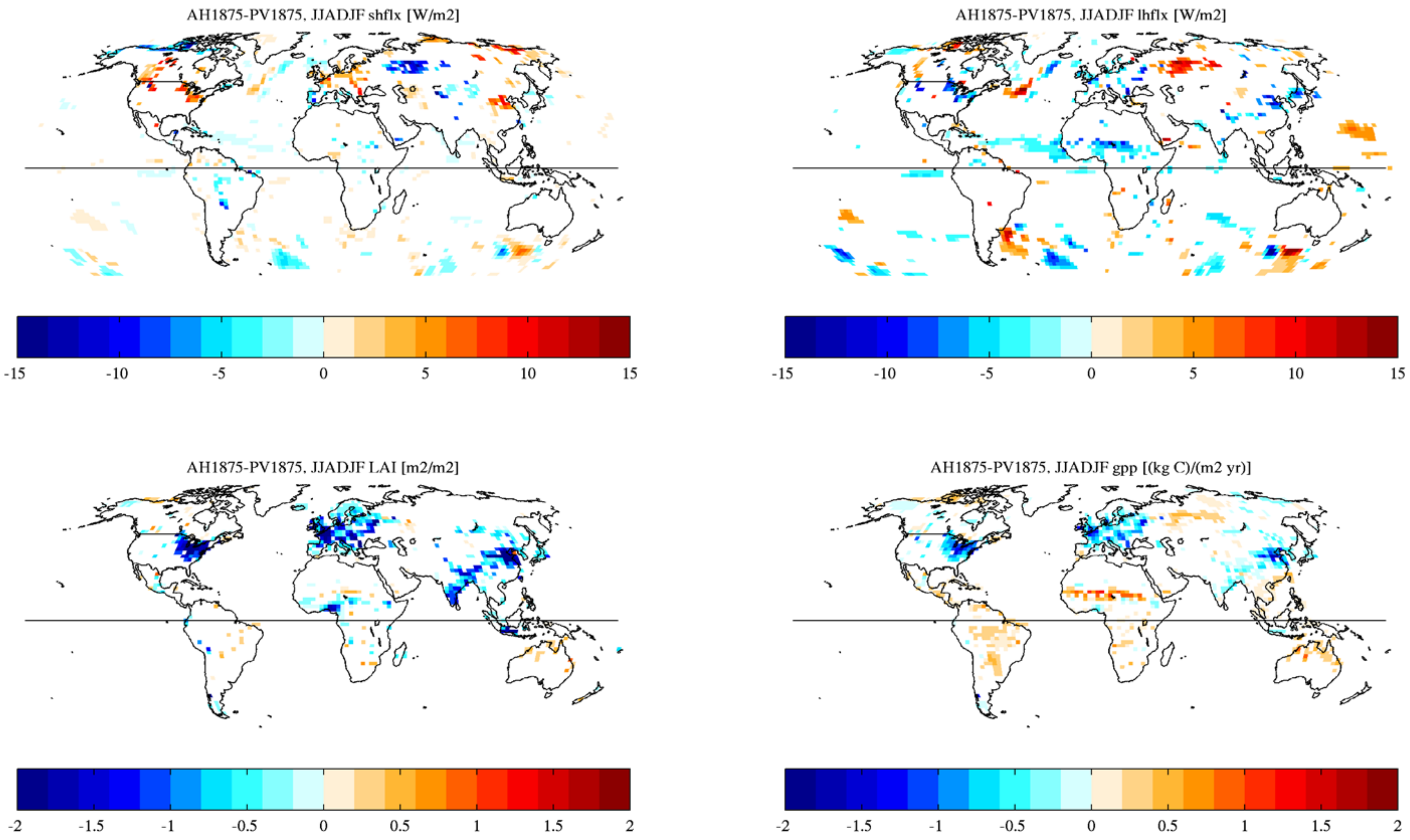

Supplementary Figure 2: Mean model response to LULCC prior to 1885. Like Figure 3, but for the time period 1861-1885. Mean differences between the AllHist and the PotVeg simulations for June-August in the northern hemisphere and for December-February for the southern hemisphere, averaged over the years 1861-1885. (a) Sensible heat flux; (b) latent heat flux; (c) LAI; and (d) gross primary productivity. Differences shown pass a standard t-test at the 90% significance level.

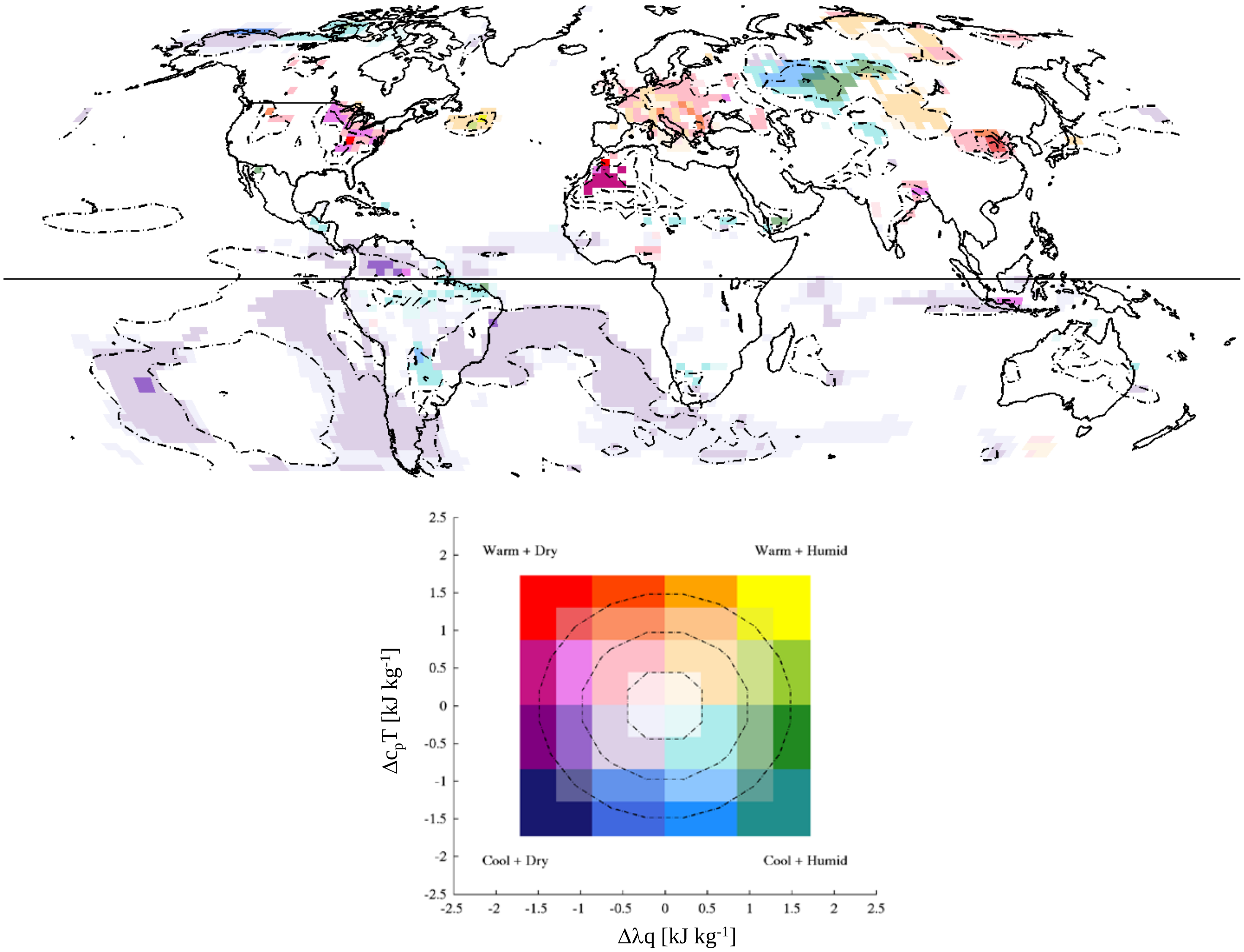

Supplementary Figure 3: Joint temperature-humidity response to LULCC prior to 1885. Like Figure 4, but for the time period 1861-1885. Mean differences (AllHist – PotVeg) for June-August in the northern hemisphere and for December-February for the southern hemisphere in (λq, c_pT) phase space, with differences of 2m values of λq and c_pT plotted according to the 2-dimensional colorgrid. q is specific humidity, λ is the latent heat of condensation, T is temperature, and c_p is the specific heat of dry air at constant pressure. Colors reflect the vector differences of the means when plotted on conserved variable diagrams like those shown in Figure 5. Differences shown have at least one of the two variables passing a modified t-test at the 90% significance level. Contours every 0.5 kJ/kg.

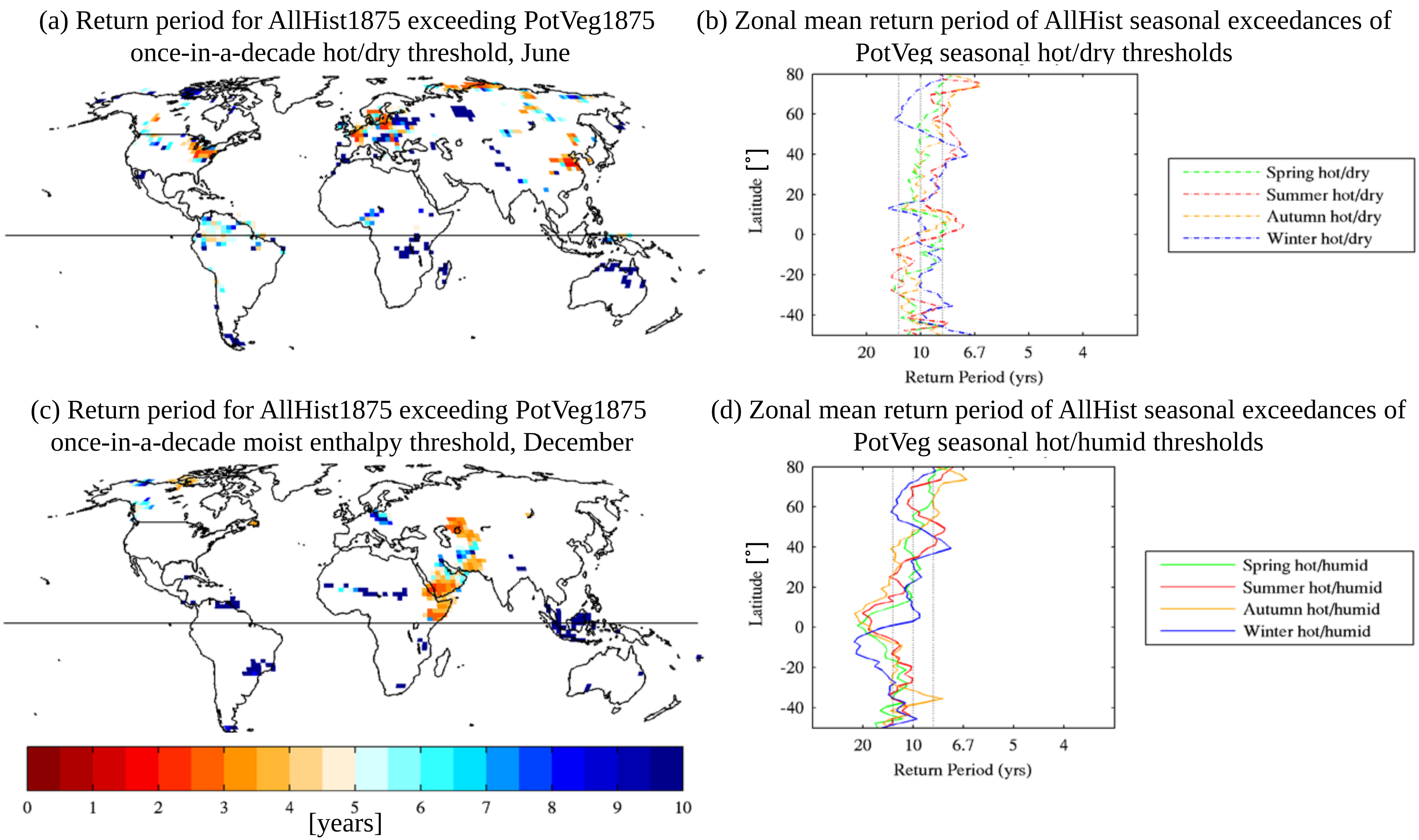

Supplementary Figure 4: Change in return periods of extreme events in response to anthropogenic LULCC. Like Figure 6, but for the time period 1861-1885. AllHist return periods for exceedance of once-in-a-decade event thresholds determined from the PotVeg run for the 1861-1885 time period for (a) the aridity threshold in June, (c) the moist enthalpy threshold in December, and seasonal, latitudinal means for (b) aridity thresholds and (d) moist enthalpy thresholds. In (a) and (c), colors are only shown where the medians of the 50-year samples are significantly different according to a Wilcoxon rank sum test at the 5% level. In (b) and (d) grey vertical lines are drawn at 6, 10, and 14-year return periods (5±2 exceedances in 50 years), with 10 years representing the null hypothesis (no change from PotVeg). These plots show that systematic differences in the frequency of these extremes between the AllHist and PotVeg simulations was not present during the first 25 years of the simulations.

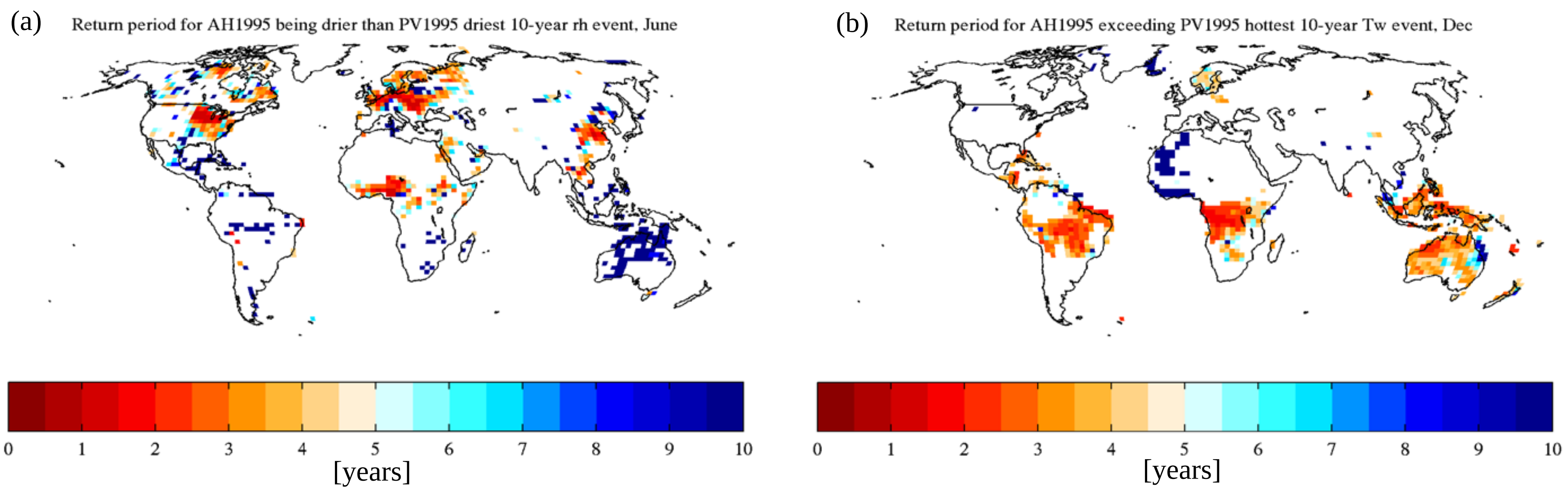

Supplementary Figure 5: Change in return periods of extreme relative humidity and wet-bulb temperature in response to anthropogenic LULCC. AllHist return periods for exceedance of once-in-a-decade event thresholds determined from the PotVeg run for the 1981-2005 time period. (a) Like Figure 6a, but for relative humidity. (b) Like Figure 6c, but for wet-bulb temperature. Colors are only shown where the medians of the 50-year samples are significantly different according to a Wilcoxon rank sum test at the 5% level.
